# Discovery of genes positively modulating treatment effect using potential outcome framework and Bayesian update

**DOI:** 10.1186/s12911-022-01852-3

**Published:** 2022-04-27

**Authors:** Youngkeun Lee, Jisoo Kim, Sung Wook Seo

**Affiliations:** 1 Department of Orthopedic Surgery, Samsung Medical Center, Sungkyunkwan University School of Medicine, Seoul, Korea; 2 Institute of Biomedical AI, Samsung Advanced Institute for Health Sciences and Technology, Sungkyunkwan University School of Medicine, Seoul, Korea

**Keywords:** Causal inference, Potential outcome framework, Genomics, Treatment modulators, Bayesian

## Abstract

**Background:**

The recent explosion of cancer genomics provides extensive information about mutations and gene expression changes in cancer. However, most of the identified gene mutations are not clinically utilized. It remains uncertain whether the presence of a certain genetic alteration will affect treatment response. Conventional statistics have limitations for causal inferences and are hard to gain sufficient power in genomic datasets. Here, we developed and evaluated a C-search algorithm for searching the causal genes that maximize the effect of the treatment.

**Methods:**

The algorithm was developed based on the potential outcome framework and Bayesian posterior update. The precision of the algorithm was validated using a simulation dataset. The algorithm was implemented to a cBioPortal dataset. The genes discovered by the algorithm were externally validated within CancerSCAN screening data from Samsung Medical Center.

**Results:**

Simulation data analysis showed that the C-search algorithm was able to identify nine causal genes out of ten. The C-search algorithm shows the discovery rate rapidly increasing until the 1500 data instances. Meanwhile, the log-rank test shows a slower increase in performance. The C-search algorithm was able to suggest nine causal genes from the cBioPortal Metabric dataset. Treating the patients with the causal genes is associated with better survival outcome in both the cBioPortal dataset and the CancerSCAN dataset which is used for external validation.

**Conclusions:**

Our C-search algorithm demonstrated better performance to identify causal effects of the genes than multiple log-rank test analysis especially within a limited number of data. The result suggests that the C-search can discover the causal genes from various genetic datasets, where the number of samples is limited compared to the number of variables.

**Supplementary Information:**

The online version contains supplementary material available at 10.1186/s12911-022-01852-3.

## Introduction

Identifying genomic sequences and analyzing data is a major focus in cancer studies [1]. An understanding of the causal relationship between therapeutic effect and genomic variances among tumors will allow individualized treatment and reduce unnecessary treatment.

There are open-access, open data sources, such as the cBioPortal for Cancer Genomics. Although a large amount of data is readily accessible to researchers, most of the identified gene mutations are not clinically utilized [2].

Conventional statistical analysis of genetic data consists of a series of single-statistic tests. The cumulative probability of false positives increases as the number of genes increases. To deal with multiple-testing problems, the false discovery rate (FDR) is used, which is “expected type I errors among the total number of rejected null hypotheses” [3]. Despite the approach, the dimension of the data significantly affects the statistical power of the test. In addition, conventional statistics only draw an association; therefore, distinguishing causal relationships from spurious associations is a challenge [4].

To draw causal effects from observational data, Rubin introduced a potential outcome framework [5, 6] Individual levels of treatment effect are derived from a comparison of two potential outcomes. however, observing the exposed and unexposed outcomes at the same time is impossible. One of the methods to overcome this fundamental problem of causal inference is to compute the potential outcome from samples matched with similar covariate profiles [4, 7, 8]. However, due to the curse of dimensionality in cancer genomics, enough sample size may not be available to match the exposed and unexposed within a genetic subset [9].

We developed an algorithm called C-search that can estimate potential outcomes using the similarity-weighted Monte Carlo method. We adopted Bayesian posterior update, which allows us to estimate the uncertainty of our decision boundary from small datasets without losing power [10]. This system was used to identify the causal genes that maximize the effect of the treatment. In this study, we compared the performance of causal gene discovery between the conventional statistical method and our C-search using a simulation dataset and an open-source gene dataset.

## Materials and methods

### Pseudo-counterfactual assumption and similarity weighted Monte Carlo

Assume that individual $i$ with variable $X_{i}$ is treated with the treatment variable $T_{i} .$ $T_{i}$ is a binary variable ($T_{i} = 1$, if treated; $T_{i} = 0$, if not). There are two potential outcomes: $Y\left( {X_{i} , T_{i} = 1} \right)$, and $Y\left( {X_{i} , T_{i} = 0} \right)$. The causal effect of the treatment can be drawn from the comparison between both [6].

Here, we define $f\left( {X_{i} } \right)$ as the outcome of an individual $i$ with variable $X_{i}$ when treated, and $g\left( {X_{i} } \right)$ as the oucome of the individual when not treated. Then, individual treatment effect ($ITE$) of the individual $i$, $ITE\left( {X_{i} } \right)$ can be written as follows:

$$ITE\left( {X_{i} } \right) = f\left( {X_{i} } \right) - g\left( {X_{i} } \right)$$

However, we can observe only one potential outcome at most [4, 8]. Therefore, we should infer the counterfactuals from an untreated data pool. We call them pseudo-counterfactuals because they are not identical to the factual.

Draw an individual $j$ with variable $X_{j}$ from the untreated data pool. Weight function $W\left( {X_{i} , X_{j} } \right)$ is defined as the probability of similarity $sim\left( {X_{i} , X_{j} } \right)$ between the factual and pseudo-counterfactual [11]. Using the similarity weighted Monte Carlo method [12], we could estimate $ITE \left( {X_{i} } \right)$ as follows:

1 $$\begin{aligned} & \mathop \sum \limits_{j} W\left( {X_{i} ,X_{j} } \right) = 1 \\ & ITE\left( {X_{i} } \right) \approx f\left( {X_{i} } \right) - \mathop \sum \limits_{j} g\left( {X_{j} } \right) \cdot W\left( {X_{i} ,X_{j} } \right) \\ \end{aligned}$$

### Measurement of the difference in survival outcome using Win probability (***Pw***_***i***_)

Survival outcome $Y$ includes survival time and survival events. Measuring individual differences in survival outcomes is difficult because they are right-censored. One of the most well-established outcome measures for survival difference is the bi-partite ranking system, such as the Wilcoxon − Mann − Whitney statistics [13]. Adopting this concept, we assumed the comparison in outcome between two individuals $i$ and $j$ as a Bernoulli trial. If $i$ lives longer than $j$, $i$ will win a score. *ITE* for the survival outcome can be defined as the win probability ($Pw_{i} :$ the chance that the treated individual $i$ wins over its untreated counterpart), which follows a binomial distribution.

The beta distribution is a conjugate prior for the binomial distribution. If we consider the comparison between two individuals $i$ and $j$ as a simple Bernoulli trial, the posterior distribution after observing the score $s$ (or observing $s$ times of winning of $i$) after $N_{j}$ trials can be defined as follows:

2 $$\begin{aligned} & Pw_{i} \sim p(``win^{\prime\prime}|X_{i} ) \\ & Pw_{i} \propto p(X_{i} |^{\prime}``win^{\prime\prime})p\left( {``win^{\prime\prime}} \right) \\ & p\left( {``win^{\prime\prime}} \right) \sim Beta \left( {\alpha_{0} , \beta_{0} } \right) \\ & Posterior\; Pw_{i} \sim Beta \left[ {\alpha_{0} + s ,\beta_{0} + \left( {N_{j} - s} \right)} \right] \\ \end{aligned}$$

### Update Win probability using similarity weighted Monte Carlo

In ideal settings where all individuals $j\left( {j \in \left\{ {1, 2, \ldots N_{j } } \right\}} \right)$ in the counterpart group that are identical to the individual $i$, the outcome of $j$ is a good estimator for the counterfactual outcome of $i.$ However, an identical condition is impossible in the observation setting. We use the similarity weighted Monte Carlo method to update $Pw_{i}$.

We matched individual $i$ with the individuals in the counterpart data pool and updated the score($s$) with the similarity weight $W\left( {i,j} \right)$ calculated from the similarity $sim\left( {i,j} \right)$ between $i$ and $j.$ The $sim\left( {i,j} \right)$ can be the Euclidean distance in the original data space [14]. Other methods use a transformed one-dimensional score, that is, a regression function, such as a propensity function [15].

Here, we defined a basal function—a regression function that approximates the survival state. The covariates of individual $i$ and matched controls $j$ are projected onto the space through the basal functions $P_{i} \left( {Y{|}X_{i} } \right)$ and $P_{j} (Y|X_{j} )$*.* To incorporate the difference onto the similarity weight, we used the Boltzmann probability distribution:

3 $$\begin{aligned} sim(i,j) & = e^{{ - |P_{i} - P_{j} |/k\tau }} \\ W\left( {i,j} \right) & = \frac{{e^{{ - \left| {P_{i } - P_{j} } \right|/k\tau }} }}{{\mathop \sum \nolimits_{j}^{N} e^{{ - \left| {P_{i } - P_{j} } \right|/k\tau }} }} \\ \end{aligned}$$

$k$ is a constant and $\tau$ is the annealing temperature. These hyperparameters represent degrees of freedom.

Let $s_{j}$ be the score from a single comparison between $i$ and $j$. The posterior distribution after observing a single comparison between $i$ and $j$ can be written as follows:

4 $$\begin{aligned} & \left\{ {\begin{array}{*{20}c} {s_{j} = 1,\; if\;Y_{i} \left( {T = 1} \right) > Y_{j} \left( {T = 0} \right)} \\ {s_{j} = 0, \;if \;Y_{i} \left( {T = 1} \right) < Y_{j} \left( {T = 0} \right)} \\ \end{array} } \right. \\ & Posterior\;Pw_{i} \sim Beta \left[ {\alpha + s_{j} \cdot W\left( {i, j} \right), \beta + \left( {1 - s_{j} } \right) \cdot W\left( {i, j} \right)} \right] \\ \end{aligned}$$

The $Pw_{i}$ is estimated from the posterior distribution after observing $N_{j}$ the number of counterpart individuals.

5 $$Posterior Pw_{i} \sim Beta\left[ {\alpha + \mathop \sum \limits_{j = 1}^{{N_{j} }} s_{j} \cdot W\left( {i, j} \right),\beta + \mathop \sum \limits_{j = 1}^{{N_{j} }} \left( {1 - s_{j} } \right) \cdot W\left( {i, j} \right)} \right]$$

We defined the observed clinical covariates as $V_{i} .$ Genetic alteration is represented by simple binary values (e.g., for each genetic profile $g_{1} ,g_{2} ,g_{3} , \ldots , g_{{N_{g} }}$, existing alteration is given a value of 1; if not, it is given a value of 0). The individual $i$ has the genetic variable $G_{i}$ that consists of a set of genetic profile.

$$\begin{aligned} & G_{i} = \left[ {g_{1} ,g_{2} ,g_{3} , \ldots , g_{{N_{g} }} } \right]_{i} \\ & g_{1} ,g_{2} ,g_{3} , \ldots , g_{{N_{g} }} \in \left\{ {0,1} \right\} \\ \end{aligned}$$

**Table:** 

Individual	Clinical covariates	Genetic covariates	$T$	$Y\left( {T = 0} \right)$	$Y\left( {T = 1} \right)$
$i$	$V_{i}$	$G_{i}$	$T_{i}$	$Y_{i} \left( {T = 0} \right)$	$Y_{i} \left( {T = 1} \right)$

To estimate $Pw_{i}$, we may use the similarity weight calculated from the basal function using clinical covariates and/or genetic covariates. We denote the weight of the basal function of clinical covariates as $W^{V} \left( {i,j} \right)$ and the weight using the basal function of the genetic covariates as $W^{G} \left( {i,j} \right)$. Using Eq. 3, $W^{V} \left( {i,j} \right)$ and $W^{G} \left( {i,j} \right)$ can be written as follows:

6 $$\begin{aligned} sim^{V} (i,j) & = e^{{ - |P_{i} (Y|V_{i} ) - P_{j} (Y|V_{j} )|/k\tau }} \\ W^{V} \left( {i,j} \right) & = \frac{{sim^{V} \left( {i,j} \right)}}{{\mathop \sum \nolimits_{j}^{{N_{j} }} sim^{V} \left( {i,j} \right)}} \\ sim^{G} (i,j) & = e^{{ - |P_{i} (Y|G_{i} ) - P_{j} (Y|G_{j} )|/k\tau }} \\ W^{G} \left( {i,j} \right) & = \frac{{sim^{G} \left( {i,j} \right)}}{{\mathop \sum \nolimits_{j}^{{N_{j} }} sim^{G} \left( {i,j} \right)}} \\ \end{aligned}$$

The win probabilities of individual $i$ using both weights are as follows:

7 $$Posterior\; Pw_{i} \sim Beta \left[ { \alpha + \mathop \sum \limits_{j = 1}^{{N_{j} }} s_{j} \cdot W^{V} \left( {i, j} \right) \cdot W^{G} \left( {i, j} \right) ,\beta + \mathop \sum \limits_{j = 1}^{{N_{j} }} (1 - s_{j} ) \cdot W^{V} \left( {i, j} \right) \cdot W^{G} \left( {i, j} \right) } \right]$$

### Causal gene suggestion

To find a single gene ($$g_{k} )$$ effect on the treatment effect, we assumed that each gene has an independent win probability $$P(``win^{\prime\prime}|g_{k} )$$.

8 $$\begin{aligned} & P(``win^{\prime\prime}| g_{k} ) \propto P \left( {g_{k} |``win^{\prime\prime}} \right) \cdot P\left( {``win^{\prime\prime}} \right) \\ & \quad k \in \left\{ {1,2,3, \ldots ,n} \right\}, n \in {\mathbb{N}} \\ \end{aligned}$$

We used the similarity weighted Monte Carlo method to estimate $P\left( {``win^{\prime\prime}|X_{i} ,g_{k} = 1 } \right)$ or individual $i$’s win probability $Pw_{i} \left( {g_{k} = 1} \right)$(Eq. 7). Observing individual $i$’s win probability $Pw_{i} \left( {g_{k} = 1} \right){ }$ updates the prior distribution of $P\left( {``win^{\prime\prime}} \right) = Beta \left( {\alpha_{0} , \beta_{0} } \right)$ and the posterior is as follows:

9 $$Posterior P\left( {``win^{\prime\prime}|g_{k} } \right) \sim Beta \left[ {\alpha_{0} + Pw_{i} \left( {g_{k} = 1} \right),\beta_{0} + \left( {1 - Pw_{i} \left( {g_{k} = 1} \right)} \right)} \right]$$

Sampling $N_{k}$-number of individuals with $g_{k} = 1$, the posterior can be as follows:

10 $$\begin{aligned} & Posterior \;P\left( {``win^{\prime\prime}|g_{k} } \right) \\ & \quad \sim Beta\left[ {\alpha_{0} + \mathop \sum \limits_{i = 1}^{{N_{k} }} Pw_{i} \left( {g_{k} = 1} \right),\beta_{0} + \left( {N_{k} - \mathop \sum \limits_{i = 1}^{{N_{k} }} Pw_{i} \left( {g_{k} = 1} \right)} \right)} \right] \\ \end{aligned}$$

In reality$, Pw_{i} \left( {g_{k} = 1} \right){ }$ is not stationary because $Pw_{i}$ depends on individual variables $V_{i} , G_{i}$. To identify the marginal treatment effect, we need to balance all confounding variables using inverse propensity score weighting (IPW) as follows.

11 $$\begin{aligned} & Posterior \;P\left( {``win^{\prime\prime}|g_{k} } \right) \\ & \quad \sim Beta\left[ {\alpha_{0} + \mathop \sum \limits_{i = 1}^{N} \frac{{Pw_{i} \left( {g_{k} = 1} \right)}}{{\hat{e}\left( {V_{i} } \right)\hat{e}\left( {G_{i} } \right)}},\beta_{0} + \left( {\mathop \sum \limits_{i = 1}^{N} \frac{{1 - Pw_{i} \left( {g_{k} = 1} \right)}}{{\left( {1 - \hat{e}\left( {V_{i} } \right)} \right)\left( {1 - \hat{e}\left( {G_{i} } \right)} \right)}}} \right)} \right] \\ & \quad \hat{e}\left( {V_{i} } \right) = P \left( {T{|}V_{i} } \right), \hat{e}\left( {G_{i} } \right) = P{(}T{|}G_{i} ) \\ \end{aligned}$$

For the treatment decision, we need to know whether treating a patient with genetic alteration $\left( {g_{k} } \right)$ is beneficial with sufficient evidence. We can estimate the posterior distribution of $Pw$ without $g_{k}$ in the same manner. If $g_{k}$ has a significant benefit for the treatment, the upper bound of the 95% confidence interval of $P(``win^{\prime\prime}|g_{k} = 0$) should be lower than the lower bound of $P(``win^{\prime\prime}|g_{k} = 1$).

## Results

### Simulation data analysis

The C-search algorithm was validated with the simulation data (Additional file 1), where the 10 causal genes that positively modulated the treatment outcome were hidden among 300 genes. We compared the precision of the C-search algorithm with that of the conventional log-rank survival analysis. Both the C-search algorithm and conventional statistics suggested 10 possible causal genes. The precision of the algorithm was determined by the number of true causal genes among the suggested genes. The performance of the algorithm was evaluated using five-fold cross-validation. To balance the covariate profile, we used propensity score matching. To perform the multiple comparison tests, we set the FDR to 0.05 and controlled it with the Benjamini − Hochberg procedure [16, 17].

**Fig. 1:**
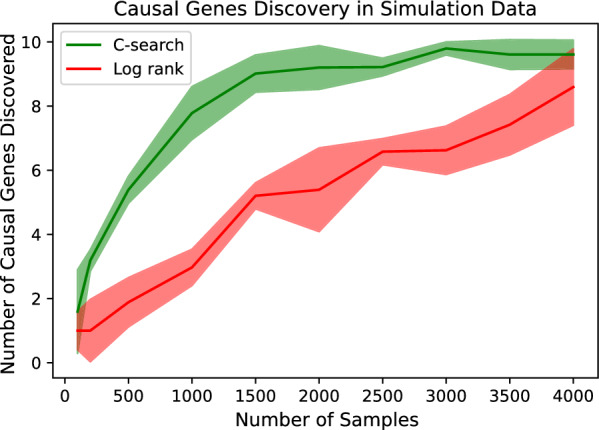
The number of causal genes discovered by C-search and conventional statistics. The X-axis is the number of samples consisting of the simulation data. The Y-axis is the number of true causal genes among the 10 suggested causal genes that the algorithm discovered. The C-search algorithm shows the discovery rate rapidly increasing until the 1500 data instances. The log-rank test shows a slower increase in performance

The power of log-rank survival analysis depends on the number of data [18]. To demonstrate whether the algorithms are dependent on the number of data, 100, 200, 500, 1000, 1500, 2000, 2500, 3000, 3500, and 4000 simulation data were used for the analysis. As the number of data increases, both algorithms show better causal gene discovery. The C-search algorithm continuously improved its discovery performance until 1500 samples were obtained and then plateaued. Conventional statistics showed a linear improvement according to the number of samples. It required at least 4000 samples to show performance comparable to that of the C-search algorithm (Fig. 1).

### Finding positive modulators from open-source data

We also analyzed the cBioPortal Metabric breast cancer data using the C-search algorithm to identify causal genes that are associated with improved outcomes of chemotherapy. The dataset includes gene mutation profiles of 173 genes and clinical data from 2433 patients with primary breast cancer [19]. Clinical data consisted of age, chemotherapy, radiation therapy, sex, survival lifetime, and survival events. Among the 2433 records, 964 records that have missing on clinical data were omitted. A total of 1,469 patients were included in the analysis (Additional file 1).

The C-search suggested nine positive modulators: *PRKCZ, CLK3, CDKN2A, BRAF, KRAS, CASP8, JAK1, PRKACG, and SIK2*. We allocated all patients who had any of them to the causal gene group and those who had not to the other gene group. If they are the true causal genes, treated patients should show a statistically significantly better prognosis compared to the untreated patients in the causal gene group. In addition, among the patients who were treated, the causal gene group should show a better prognosis than the other gene group.

**Fig. 2:**
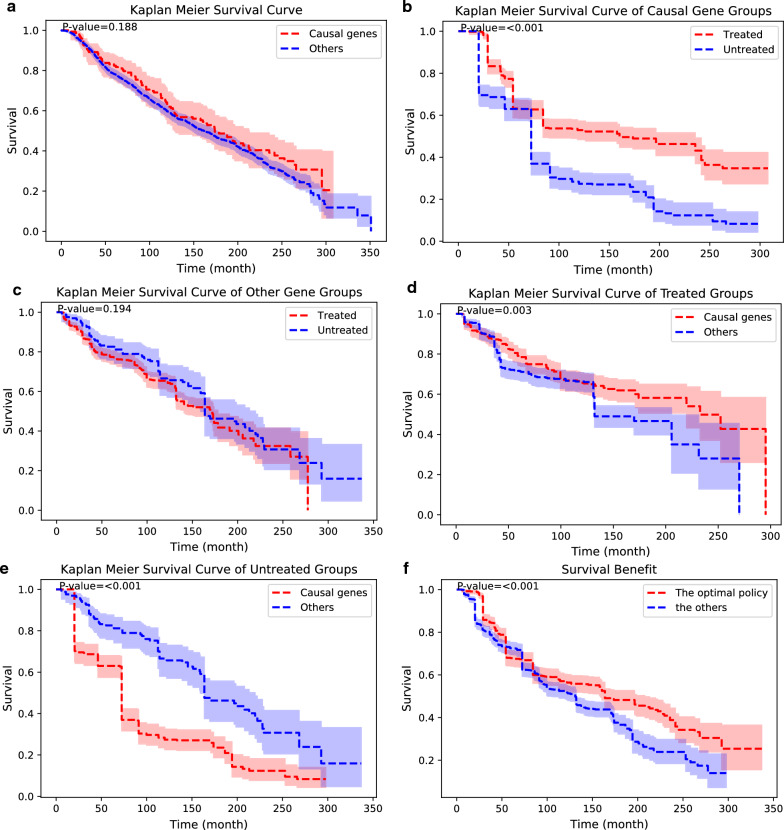
Discovery of positive modulator genes by C-search in the cBioPortal breast cancer dataset. Nine causal genes are discovered, and patients with causal genes are assigned to the causal gene group. Patients without casual genes are assigned to the other gene group. All Kaplan − Meier survival curves are adjusted with propensity score matching [39]; 95% confidence intervals are depicted, and p-values are noted. **a** Kaplan − Meier survival curves of the causal gene group and the other gene group. **b** Treated and untreated patients are compared in the causal gene group. **c** Treated and untreated patients are compared for the other gene group. **d** The causal gene and other gene group are compared between treated patients. **e** The causal gene and other gene group are compared between the untreated patients. **f** Survival curve following the optimal policy and the other policy is shown

There were no overall survival differences between the causal gene group and the other gene group (Fig. 2a). Among patients with causal genes, the treatment group showed a better prognosis than the untreated group (Fig. 2b). Meanwhile, in the other gene group, there were no statistically significant differences between the treated and untreated groups (Fig. 2c). Among the treated patients, the causal gene group showed significantly better survival than the other gene group (Fig. 2d). In the untreated group, the other gene group showed better survival outcomes compared with the causal gene group (Fig. 2e). We define the optimal policy as treating patients with causal genes and not treating patients without causal genes. The other policy is to treat patients in the other gene group and not to treat patients in the causal gene group. The Kaplan − Meier survival curve of the optimal policy showed better survival outcomes than the other policies (Fig. 2f).

### Conventional log-rank analysis of open-source data

To discover causal genes using conventional statistics from the cBioPortal dataset, we performed log-rank survival analysis. As the dataset includes gene mutation profiles of 173 genes, multiple log-rank survival analyses were applied for each mutation profile. For each gene mutation, the patients treated were divided into two groups: one group consisted of patients who harbored the mutation and one group of patients without the mutation. Survival outcomes were compared between the two groups. Propensity score matching was used to balance the covariate profile. We set the FDR to 0.01 and controlled it using the Benjamini − Hochberg procedure [16, 17].

**Fig. 3:**
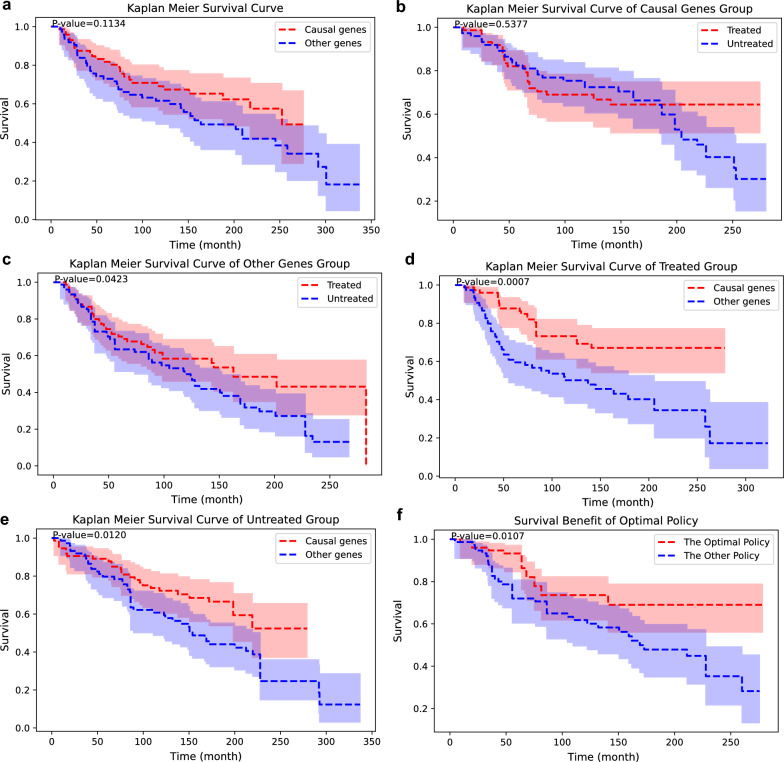
Discovery of positive modulator genes using conventional log-rank analysis in the cBioPortal breast cancer dataset. Ten causal genes are discovered, and patients with causal genes are assigned to the causal gene group. Patients without casual genes are assigned to the other gene group. All Kaplan − Meier survival curves are adjusted with propensity score matching [39]; 95% confidence intervals are depicted, and p-values are noted. **a** Kaplan − Meier survival curves of the causal gene group and the other gene group. **b** Treated and untreated patients are compared in the causal gene group. **c** Treated and untreated patients are compared for the other gene group. **d** The causal gene and other gene group are compared between the treated patients. **e** The causal gene group and the other gene group are compared between the untreated patients. **f** Survival curve following the optimal policy and the other policy is shown

A total of 10 genes were shown to be correlated with positive treatment outcomes: *EGFR, CLK3, PTEN, CDH1, GATA3, KRAS, RB1, PRKACG, NEK1*, and *NRAS*. Patients who have any of these are allocated to the causal gene group, and those who do not are assigned to other gene group. Kaplan − Meier survival curves comparing the overall survival differences between both groups are depicted in Fig. 3a. No survival differences were observed between patients with causal genes and without causal genes. In both the causal gene group and the other gene group, treatment was not associated with positive outcomes compared with no treatment (Fig. 3b, c). Meanwhile, in the treated group, the causal gene group showed better survival than the other gene group (Fig. 3d). Among the untreated patients, the causal gene group and the other gene group had no statistically significant differences in survival (Fig. 3e). Following optimal policy demonstrated better survival than following the other policy (Fig. 3f).

### Comparison between C-search and conventional log-rank analysis

**Fig. 4:**
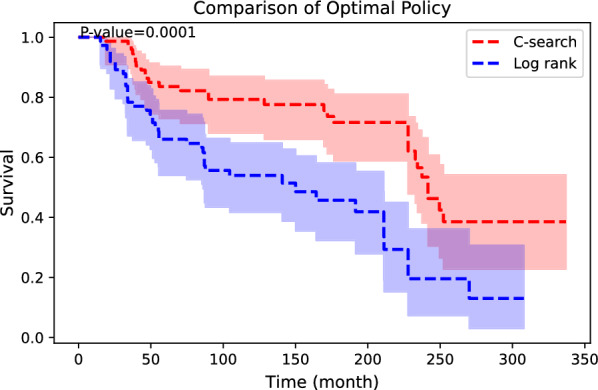
Survival differences between the optimal policy determined by C-search and conventional log-rank analysis are shown. The C-search’s policy shows better outcomes than the others. The Kaplan − Meier survival curve is adjusted with propensity score matching; 95% confidence intervals are depicted, and *p*-values are noted

We compared the Kaplan − Meier survival curve of the following optimal policy, which was determined by the genes that each algorithm discovered. The survival outcome following the C-search policy showed statistically significant better survival outcomes (Fig. 4).

### Validation with external data

To determine whether the genes found by the algorithm will act as causal genes in the other dataset, we used CancerSCAN screening data from Samsung Medical Center. CancerSCAN is a custom panel developed by the Samsung Genomic Institute [20]. The usage of the data was approved by the institutional review boards of the participating institutions (Samsung Medical Center 2019-11-127).

CancerSCAN data consists of 559 breast cancer samples obtained at the Samsung Medical Center from January 2014 to September 2016. Mutation profiles of 81 genes and clinical data on age, chemotherapy, radiation therapy, sex, survival time, and survival events were included (Additional file 1). Among the causal genes suggested by C-search, mutation profiles of *BRAF, KRAS, CDKN2A*, and *JAK1* were found in the CancerSCAN dataset. We assigned patients to the C-search causal gene group who acquired at least one of the mutations. The *CDH1, EGFR, KRAS, PTEN*, and *RB1* are genes suggested by log-rank analysis whose mutation profiles exist in the CancerSCAN dataset. Patients with these mutations are assigned to the conventional statistics causal gene group.

**Fig. 5:**
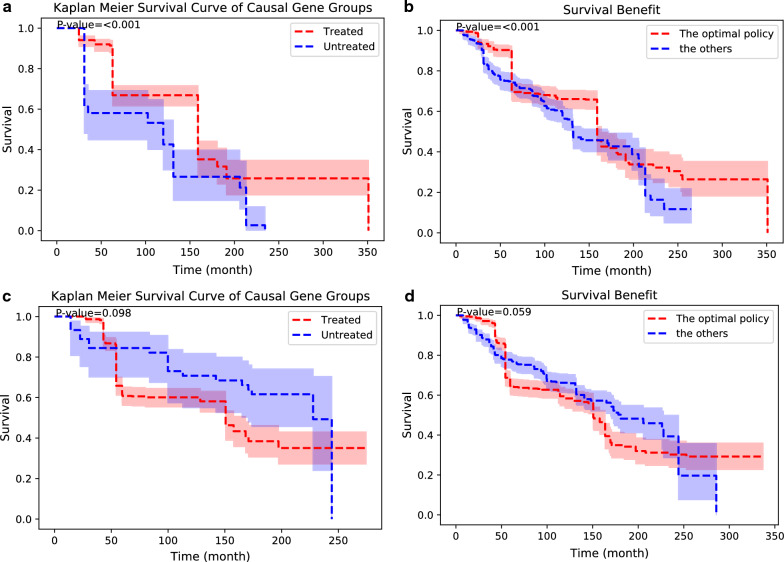
External validation of the causal genes suggested by C-search and conventional log-rank analysis. **a** Kaplan − Meier survival curve of the treated and the untreated among the C-search causal gene group. **b** Kaplan − Meier survival curve following and not following C-search optimal policy. **c** Kaplan − Meier survival curve of the treated and the untreated among the conventional statistics causal gene group. **d** Kaplan − Meier survival curve following and not following conventional statistics optimal policy

Figure 5a shows the significant survival difference between the treated and the untreated in the C-search causal gene group. The optimal policy suggested by the C-search showed a significantly better survival outcome (Fig. 5b). Meanwhile, among conventional statistical causal gene group, the treated did not demonstrate statistically better survival compared to the untreated (Fig. 5c). The survival outcomes following the optimal policy suggested by conventional statistics and those of the other policies are not statistically different (Fig. 5d).

## Discussion

Biotechnological breakthroughs in gene profiling have led to an increased focus on individualized precision therapy [21]. Clinicians want to treat patients who will benefit the most from the therapy while avoiding treatment that will not benefit or even get harm from therapy.

There are studies on genetic assays to predict prognosis or response to treatment for breast cancer [22]. BluePrint molecular subtyping profile uses 80 genes to determine the sensitivity to adjuvant treatment [23]. Prosigna Breast Cancer Prognostic Gene Signature Assay utilizes the PAM50 test, which identifies gene signatures specific to breast cancer subtypes (luminal A/B, HRE2, basal-like) [24]. Both studies used genomic profiles to infer the molecular subtypes of cancer and drew a correlation between the subtypes and the prognosis or response to treatment. However, since the correlation does not infer causation, we cannot conclude that the specific genomic profile results in a positive response to treatment. In contrast, our C-search algorithm uses a potential outcome framework to study the causal effect of each gene on treatment.

The estimation of causal effects from observational studies can be done in a number of ways. Methods based on propensity scores match, stratify, and/or inversely weight covariates that affect treatment allocation [25, 26]. G-computation implements regression models [27]. Mendelian randomization uses germline gene mutations as instruments to make causal inference [28, 29]. These methods are used to estimate average treatment effect of the target population. To estimate and compare the treatment effect of the patient who have a specific genetic mutation, one must calculate conditional causal effect, which is the average treatment effect of a subgroup of patients with that mutation [30]. However, due to the curse of dimensionality in cancer genomics, some subgroups may lack sufficient sample size to draw meaningful conclusions [31]. In addition, when the algorithm handles a smaller dataset, the result drawn from the inference has a considerable amount of uncertainty. It is important to incorporate uncertainty into the prediction of the algorithm [32, 33]. The C-search algorithm updates the gene’s win probability with a Bayesian update, thus, reflecting its uncertainty in the analysis. Pseudocode for the algorithm and the computational complexity are shown in Additional file 1.

We demonstrated that the C-search algorithm can identify causal genes from a simulation dataset that includes hidden confounders. Compared to conventional log-rank analysis, C-search requires fewer data to gain sufficient power to find the causal genes. Therefore, the C-search may find more candidate causal genes than conventional association studies using genomic data. When there are not enough data subsets, it is important as we used Bayesian update to estimate the gene’s win probability.

Both C-search and log-rank analysis successfully found positive modulators in the Metabric dataset (Figs. 2, 3), yet the gene set found by C-search showed better results than log-rank analysis (Fig. 4). Among the positive regulators that C-search and conventional log-rank analysis found, two genes are found in common in both algorithms: *KRAS* and *PRKACG*. The *KRAS* gene targets several miRNAs to enhance chemotherapy in acute myeloid leukemia, lung cancer, breast cancer, and gallbladder cancer [34–36]. *PRKACG* is a gene that encodes the protein kinase A subunit Cγ, whose role in cancer has not been elucidated. The causal genes suggested by both algorithms contained relatively few overlapping genes. This may be due to intercorrelated genes, at least to some extent; therefore, one of the co-expressed genes may be selected for the set as a predictor and yield comparable results [37].

Our study has several limitations. There were only a few clinical variables available in the open-source dataset; therefore, hidden confounders may affect the performance of the algorithm. IPW may result in bias in this setting [15, 38]. However, the algorithm performance in the simulation data, including 10 hidden confounders, showed better results than log-rank analysis. As there are no golden standard datasets to evaluate the performance of the algorithm to determine the treatment modulating effect of genetic variables, we can only evaluate the algorithm’s performance indirectly. Using the CancerSCAN dataset, we were able to externally validate that the causal genes suggested by C-search showed comparable results in the external dataset.

## Conclusion

We proposed an algorithm that uses a potential outcome framework and Bayesian updating, inferring the causal effect of the genetic variable on treatment outcomes. The proposed algorithm was shown to find causal genes from the simulation data in a relatively small number of samples compared to the log-rank analysis. It also showed its performance in finding positive treatment modulators from the open-source breast cancer dataset, which is validated with external data. The C-search algorithm may be applied to various types of datasets where the number of samples is limited compared to the number of variables.

## Supplementary Information

**Additional file 1**. How to simulate data generation, demographics of cBioPortal dataset, demographics of CancerSCAN dataset, pseudocode of the C-search Algorithm, computational complexity 

## Data Availability

The cBioPortal dataset analyzed during the current study is available from the cBioPortal repository, https://cbioportal-datahub.s3.amazonaws.com/brca_metabric.tar.gz. The Samsung Medical Center CancerSCAN dataset is not publicly available as it is not approved to be open in public, but it is available upon request from the corresponding author for academic use. The code for the algorithm is available upon request from the corresponding author for academic use. All implementation details are described in the Methods section so that they can be replicated with nonproprietary libraries.

## Abbreviations

FDR: False Discovery Rate
ITE: Individaul Treatment Effect
IPW: Inverse Propensity Score Weighting
