# Correction: Discovery of genes positively modulating treatment effect using potential outcome framework and Bayesian update

**DOI:** 10.1186/s12911-025-03141-1

**Published:** 2025-09-08

**Authors:** Youngkeun Lee, Jisoo Kim, Sung Wook Seo

**Affiliations:** 1 Department of Orthopedic Surgery, Samsung Medical Center, Sungkyunkwan University School of Medicine, Seoul, Korea; 2 Institute of Biomedical AI, Samsung Advanced Institute for Health Sciences and Technology, Sungkyunkwan University School of Medicine, Seoul, Korea

**Correction: BMC Medical Informatics and Decision Making (2022) 22:113**

10.1186/s12911-022-01852-3

Following publication of the original article [1], we were notified of a spelling error in the first author’s name.

Originally published name: Young Keun Lee.

Corrected name: Youngkeun Lee.

The original article has been corrected.

## References

[1] Lee, et al. Discovery of genes positively modulating treatment effect using potential outcome framework and Bayesian update. BMC Med Inform Decis Mak. 2022;22:113. 10.1186/s12911-022-01852-3 10.1186/s12911-022-01852-3 PMC9047392 35477453

