# Supplementary material for: Discovery of genes positively modulating treatment effect using potential outcome framework and Bayesian update
Source: BMC Med Inform Decis Mak. 2022 Apr 27;22:113. doi: 10.1186/s12911-022-01852-3 (PMC9047392; doi:10.1186/s12911-022-01852-3)
Supplement: Supplementary file 1 — Additional file 1. How to simulate data generation, demographics of cBioPortal dataset, demographics of CancerSCAN dataset, pseudocode of the C-search Algorithm, computational complexity [file 12911_2022_1852_MOESM1_ESM.docx]

## Supplementary

## 1. Simulation data generation

We built the simulation data consisting of the binary treatment variable $T_{i}$, 10 clinical variables $X_{i}$, 10 hidden clinical variables $A_{i}$, 300 genetic variables $G_{i}$ and survival outcomes $Y_{i}$.

$${x_{i}^{k}\in X}_{i}, k=1\sim10$$

$${a_{i}^{k}\in A}_{i}, k=1\sim10$$

$${g_{i}^{k}\in G}_{i}, k=1\sim300$$

Each gene from $G_{i}$ is allocated as 1 with a probability of 0.9 and as 0 with a probability of 0.1. Each clinical variable $x_{i}^{k}$and hidden variable $a_{i}^{k}$ are sampled from a folded random normal distribution with mean $\mu= 0$and standard deviation $\sigma=3$. We assumed that six of the 10 clinical variables were affected by genetic variables.

$$x_{i}^{k}:=x_{i}^{k}+W_{x}^{T}\cdot G_{i}+ u_{x} (k=1, 2, 3, 4, 5, 6)$$

$$W_{x}\sim U\left( 0, 2 \right), u_{x}\mathbb{\sim N}\left( 0, 1 \right)$$

$$a_{i}^{k}:=a_{i}^{k}+W_{a}^{T}\cdot G_{i}+ u_{a} (k=1, 2, 3, 4, 5, 6)$$

$$W_{a}\sim U\left( 0, 2 \right), u_{a}\mathbb{\sim N}\left( 0, 1 \right)$$

where $W_{x}$, $W_{a}$ is a set of parameters sampled from a uniform distribution, and $u_{x}, u_{a}$ is a random variable sampled from a normal distribution.

The treatment assignment probability $p_{i}$ is a probability associated with $X_{i}$ and $G_{i}^{c}$, where $G_{i}^{c}$ is a confounder gene that may be directly or indirectly associated with the treatment probability through latent paths other than $X_{i}$. We chose 10 genes ($g_{i}^{k}, k=154, 155, \cdots, 163$) as $G^{c}$. $T_{i}$ is a binary variable that takes the value of 1 with probability $p_{i}$. $W_{1}^{'}$ and $W_{2}^{'}$ are the sets of parameters sampled from a uniform distribution, and $u'$ is a random variable sampled from the normal distribution:

$p_{i}=\sigma\left( {W_{1}^{'T} \cdot X}_{i}+ W_{2}^{'T}\cdot G_{i}^{C}+ u^{'} \right)$

$$\left\{ \begin{aligned} T_{i}=1 \sim p_{i} \\ T_{i}=0 \sim(1-p_{i}) \end{aligned} \right.$$

$$W^{'} \sim U\left( 0,2 \right), u^{'}\mathbb{\sim N}\left( 0,1 \right) , \sigma\left( x \right)=1/(1+e^{-x})$$

We assume that there are causal genes ($G^{*}$) that affect the individual treatment effect ($ITE)$. Ten hidden causal genes ($g_{k}, k=1, 2, \cdots, 10$) were set to have a linear correlation as follows:

$$ITE \left( G_{i}^{*} \right)=W^{''T}\cdot G_{i}^{*}$$

$$W^{''} \sim U\left( 0,2 \right)$$

where $W^{''}$ is a set of parameters sampled from a uniform distribution.

$Y_{i}$ is an individual survival outcome influenced by $X_{i}$ and $G_{i}$. $T_{i}$ also has an additive effect on survival time through an individual mechanism ($ITE$), as follows:

$$Y_{i}=e^{{(A}^{T}X_{i}+{B^{T}\cdot G_{i}+ ITE}_{i} \cdot T)}+0.01u$$

$A \sim U\left( -2,2 \right)$, $B \sim U\left( -2,2 \right)$

A and B are sets of parameters sampled from a uniform distribution.

Using these equations, we generated $N$ individual data set$\mathcal{D=}\left\{ (X_{i}, {G_{i}, T}_{i}, Y_{i}) \right\}_{i=1}^{N}$.

**2. Demographics of cBioPortal dataset**

| Population size | | 1469 |
| --- | --- | --- |
| Mean follow up time (months) | | 127.71 |
| Event (Death) |  | 826 |
| Mean age |  | 60.65 |
| Chemotherapy |  | 320 |
| Radiotherapy |  | 960 |
| Stage | 0 | 12 |
|  | 1 | 501 |
|  | 2 | 828 |
|  | 3 | 118 |
|  | 4 | 10 |

**Table 1** Demographics of cBioPortal dataset. Total 1469 samples are included in analysis.

**3. Demographics of CancerSCAN dataset**

| Population size | | 559 |
| --- | --- | --- |
| Mean follow up time (months) | | 65.76 |
| Event (Death) |  | 50 |
| Mean age |  | 41.96 |
| Chemotherapy |  | 499 |
| Radiotherapy |  | 234 |
| Stage | 0 | 0 |
|  | 1 | 115 |
|  | 2 | 231 |
|  | 3 | 142 |
|  | 4 | 37 |
|  | NA | 34 |

**Table 2** Demographics of CancerSCAN dataset. Total 559 samples are included in analysis.

**4. Pseudocode of the C-search Algorithm**


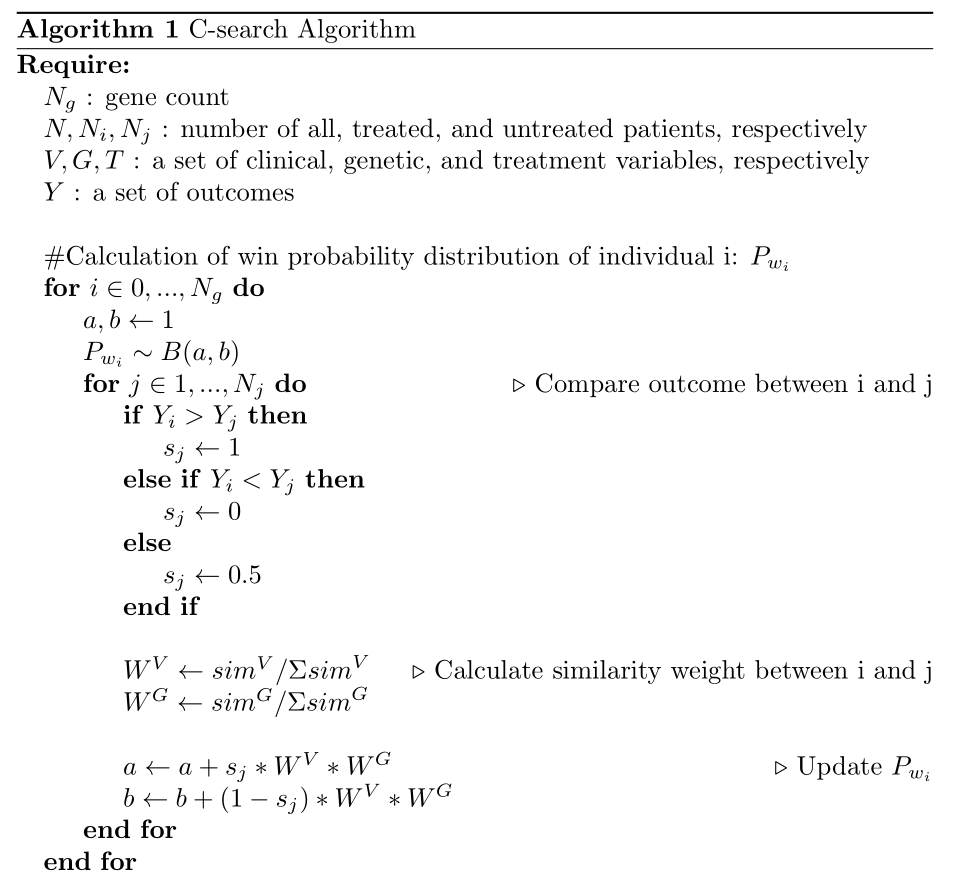


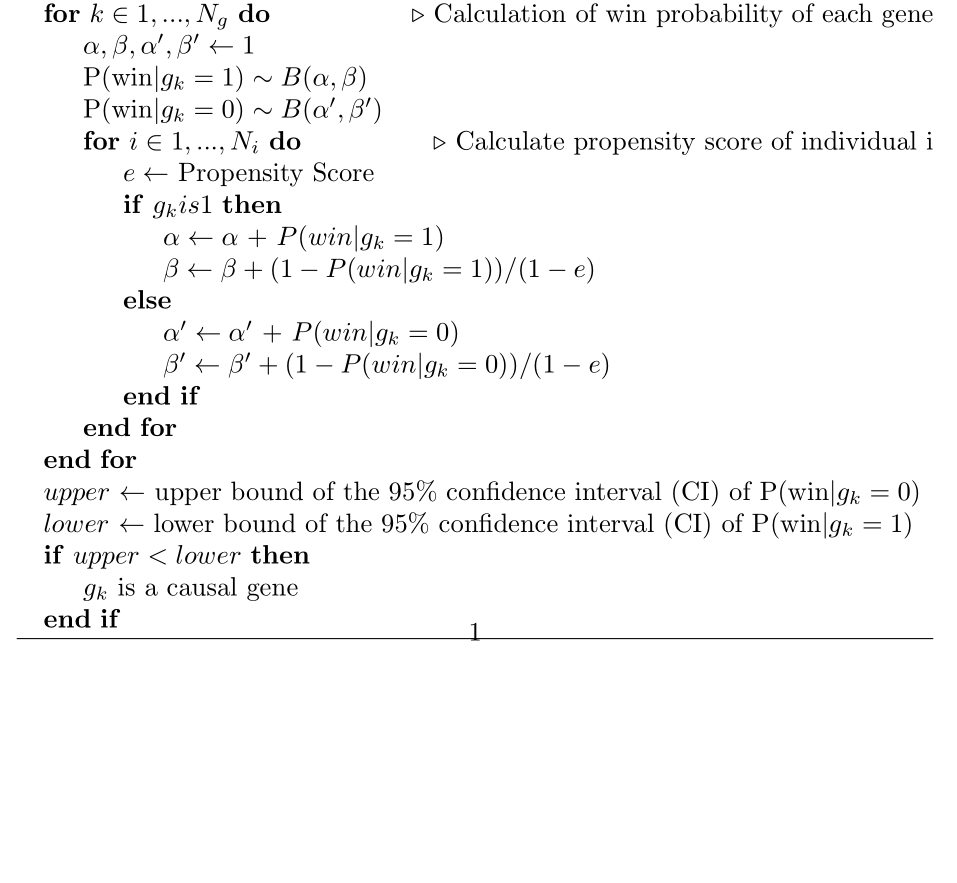


Computational Complexity: $O(N_{g}*N)$
